# Left ventricular ejection fraction estimation using mutual information on technetium-99m multiple-gated SPECT scans

**DOI:** 10.1186/s12938-015-0117-2

**Published:** 2015-12-23

**Authors:** Shih-Neng Yang, Shung-Shung Sun, Geoffrey Zhang, Kuei-Ting Chou, Shih-Wen Lo, Yu-Rou Chiou, Fang-Jing Li, Tzung-Chi Huang

**Affiliations:** Department of Biomedical Imaging and Radiological Science, China Medical University, No. 91, Hsueh-Shih Road, Taichung, 40402 Taiwan; Department of Radiation Oncology, China Medical University Hospital, No. 2, Yude Road, Taichung, 40447 Taiwan; Department of Nuclear Medicine and PET Center, China Medical University Hospital, No. 2, Yude Road, Taichung, 40447 Taiwan; Department of Radiation Oncology, Moffitt Cancer Center, 12902 Magnolia Drive, Tampa, FL 33612 USA; Department of Radiology, Taipei Municipal Wanfang Hospital, No.111, Section 3, Hsing-Long Rd, Taipei, 116 Taiwan; Department of Radiation Oncology, Tri-Service General Hospital, No. 325, Section 2, Chenggong Rd., Neihu District, Taipei City, 114 Taiwan; Department of Bioinformatics and Medical Engineering, Asia University, No. 500, Lioufeng Road, Wufeng, Taichung 41354 Taiwan

**Keywords:** MUGA, Mutual information, LVEF

## Abstract

**Background:**

A new non-linear approach was applied to calculate the left ventricular ejection fraction (LVEF) using multigated acquisition (MUGA) images.

**Methods:**

In this study, 50 patients originally for the estimation of the percentage of LVEF to monitor the effects of various cardiotoxic drugs in chemotherapy were retrospectively selected. All patients had both MUGA and echocardiography examinations (ECHO LVEF) at the same time. Mutual information (MI) theory was utilized to calculate the LVEF using MUGA imaging (MUGA MI).

**Results:**

MUGA MI estimation was significantly different from MUGA LVEF and ECHO LVEF, respectively (p < 0.005). The higher repeatability for MUGA MI can be observed in the figure by the higher correlation coefficient for MUGA MI (r = 0.95) compared with that of MUGA LVEF (r = 0.80). Again, the reproducibility was better for MUGA MI (r = 0.90, 0.92) than MUGA LVEF (r = 0.77, 0.83). The higher correlation coefficients were obtained between proposed MUGA MI and ECHO LVEF compared to that between the conventional MUGA LVEF and ECHO LVEF.

**Conclusions:**

MUGA image with the aid of MI is promising to be more interchangeable LVEF to ECHO LVEF measurement as compared with the conventional approach on MUGA image.

## Background

Left ventricular ejection fraction (LVEF) is an important indicator in left ventricular dysfunction diagnosis. Electrocardiographically (ECG) gated myocardial perfusion on single-photon emission tomography (SPECT) and multigated acquisition (MUGA) is an effective method for measuring left ventricular function in patients over a wide range of left ventricle (LV) volumes and LVEF values [1]. Magnetic resonance imaging (MRI) and echocardiography (ECHO) are alternatives with non-ionizing-radiation imaging for LVEF estimation. However, MRI usually takes a long scan time, and is associated with high cost and often unavailable in many smaller hospitals. On the other hand, ECHO is rather preferred clinically because of the straightforward preparation for LVEF examinations and its higher accuracy.

Cardiotoxicity could be induced by the drugs of chemotherapy (i.e. Anthracyclines). It may give rise to serious heart failure. Therefore, the oncology patient who receives chemotherapy will have exams before and after the course to detect a possible subclinical decline in left ventricular function. When such a decline occurs, the chemotherapy course is adjusted to limit cardiotoxicity. Currently, MUGA imaging is one of the most widely used methods to assess left ventricular function in patients who receive chemotherapy with risk of the cardiotoxicity associated with the drugs [2, 3]. However, LVEF measured by MUGA imaging varies depending on both the acquisition parameters, including SPECT scan timing, frame number, the use of collimators, etc., and the processing method used for analysis, and there are no standard evidence-based guidelines currently available for patients monitoring [4]. Generally, a measured LVEF value greater than 50 % with MUGA scanning is considered normal and a drop in LVEF by greater than 10 % is consistent with early cardiotoxicity, and the chemotherapeutic drug is usually discontinued immediately [5]. MUGA imaging for LVEF estimation was shown to have inter- and intra- observer variations and also varies widely between centers and computer processing systems [6, 7]. The low correlation for LVEF measurement between the MUGA and echocardiography examinations is also reported in a previous study [6]. LVEF value using MUGA examination is determined by the linear difference of counts in end-diastolic volume (EDV) and end-systolic volume (ESV) clinically. However, the inherent sources, including low count density, partial volume effect and incorrect background subtraction, tend to cause errors in LVEF estimation using MUGA imaging if linear calculation is applied. Currently, ECHO is the most accurate method for LVEF analysis, although it also depends on the operator’s skill and it is time consuming.

Mutual information (MI) has been used to quantify the similarity with images, and the MI value represents entropy-based image similarity invariant to the overlapped region of two images [8–10]. In this study, a new non-linear approach based on MI theory was applied to calculate the LVEF by clinical MUGA imaging (MUGA MI). The MUGA MI estimation was compared with the conventional MUGA LVEF in terms of the repeatability and the reproducibility. In addition, the results of MUGA MI were compared with the estimation of ventricular ejection fraction by echocardiography. This new non-linear approach (MUGA MI) aimed to lower inter-observer variation and better repeatability compared to the conventional approaches on MUGA image.

## Methods

### Patients

The study group included all patients who underwent both gated planar left ventriculography (MUGA) and echocardiography examinations for LVEF measurements in our hospital from August 2012 through July 2013. Fifty patients (18 male and 32 female, mean age 61, range 33–91 years) were retrospectively selected in this study for the estimation of the percentage of LVEF (MUGA LVEF %) monitoring the effects of various cardiotoxic drugs in chemotherapy. Written informed consent was obtained from all patients, and the collection of clinical patient data in this study was approved by the institutional review board of China Medical University Hospital (DMR99-IRB-010-2).

### MUGA Imaging

A Tc-99m MUGA scan was taken with Tc-99m pertechnetate- labeled autologous red blood cells (RBCs). Approximately 740 MBq of Tc-99m was used for the labeling of RBCs in each case. Planar acquisition was done by using a γ-camera (Infinia Hawkeye 4, General Elaectric company, USA) equipped with a high-resolution collimator. A Tc-99 m MUGA scan was acquired over 600 cardiac cycles with 24 frames per R–R interval (20 % window setting, left anterior oblique position at 45º), and the %LVEF was automatically calculated by drawing a region of interest over LV end diastolic volume (EDV) and LV end systolic volume (ESV). The count parameter of our study was 200,000 per frame (minimum). The spatial resolution of MUGA images was 64 × 64 and FOV was 282.85 × 282.85 mm^2^. The scan time of MUGA scan was about 15 min.

### Echocardiography

**Fig. 1:**
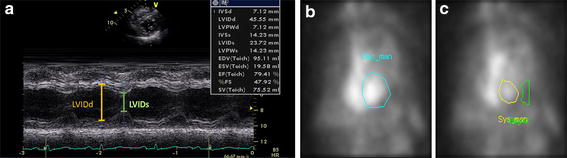
An example of LVEF estimation based on **a** the *LVIDs* and the *LVIDd* using ECHO **b** EDV and **c** ESV using MUGA LVEF and MUGA MI

Two dimensional echocardiography with M-mode was performed by one experienced operator. A standardized imaging protocol was adopted with cross-sectional imaging of the left ventricle immediately distal to the mitral valve tips and apical two-dimensional imaging based on orthogonal four- and two-chamber views. M-mode left ventricular ejection fraction (ECHO LVEF) based on the cubed method was calculated as $\frac{EDV \,- \,ESV}{ESV}$, where $EDV = \frac{{7\, \times \,(LVIDd)^{3} }}{{\left[ {2.4 \,+ \,(LVIDd)} \right]}}$ and $ESV = \frac{{7 \,\times\, (LVIDs)^{3} }}{[2.4 \,+\, (LVIDs)]}$ (LVIDd = left ventricular internal diastolic, LVIDs = left ventricular internal systolic). Volumes were calculated from three cardiac cycles disregarding ectopic and postectopic beats in the derivation of LVEF. An example of LVEF estimation based on the *LVIDs* and the *LVIDd* using ECHO is shown in Fig. 1a.

### MUGA mutual information

Mutual information (MI) is an important concept in information theory [8, 9], which has been used to measure the statistical dependence between two random variables, or the amount of information between two objects such as images. MI is usually derived from joint probability or entropy of the image feature interpreted as a measure of uncertainty, variability, or complexity. If the information correlation between two objects is small, the two objects are likely to be independent. Otherwise, they are dependent. It turns out that the joint probability between the features of two objects plays a crucial role in determining the feature correspondences, or homologies, and the outliers, or non-homologies. The equation defining the MI used in this study is given by [10]:

$$MI(U,V) = \sum\limits_{u} {\sum\limits_{v} {\frac{h(u,v)}{N}\log } } \frac{{\frac{h(u,v)}{N}}}{{\frac{h(u)}{N}*\frac{h(v)}{N}}}$$, where $$h(u,v)$$: joint histogram of $$u$$ and $$v$$$$h(u)$$, $$h(v)$$: histogram of $$u$$ and $$v$$ respectively, $$N$$: the number of units (pixels in this study) in $$u$$ and $$v$$.

In this study, an in-house MI based software was applied on MUGA image intensity statistics (MUGA MI) to estimate the LVEF which is defined as

$$LVEF \, MI(\% ) = \frac{MI(EDV, \, ESV)}{MI(EDV, \, EDV)}$$

where *MI(EDV, ESV)* computes the joint probability histogram of the EDV and ESV, and *MI(EDV, EDV)* is the maximum MI value in MUGA images. LVEF MI % is expressed as a percentage of heart pumps between EDV and ESV using maximum MI value as the reference. In fact, LVEF MI % is a measure of how much blood is being pumped out of the left ventricle of the heart with contraction.

### Intra- and inter-operator variability

The comparison of intra- and inter-operator observability are based on the results measured from different region of interest (ROI) delineated by physicians. Following the routine clinical practice, all the ROIs for MUGA LVEF and MUGA MI measurement was manually delineated by two independent experienced physicians (A, B) for assessments of the inter-operator variability. Physician A performed the ROI delineation twice at different times for assessments of the intra-operator variability, from which A1 and A2 represent the ROI of 1st and 2nd times, respectively. Examples of ROIs for MUGA LVEF and MUGA MI measurements calculated from EDV, ESV and background are showing in Fig. 1b, c.

### Data analysis

Taking ECHO as the reference, the aim of the evaluations was to pick the one, either MUGA LVEF or MUGA MI, that better correlates with ECHO. To do this, correlation coefficient (r) between ECHO and MUGA LVEF or MUGA MI was calculated using least-square fit. Statistical analysis was conducted using the two tailed t test and the results were considered significant at p < 0.05. The variability of the results generated by the two operators was assessed using the standard deviation of the differences between the two results. All of the statistical analysis was performed using SPSS 16.0 (IBM SPSS Statistics; IBM Corporation, Armonk, NY, USA).

## Results

**Fig. 2:**
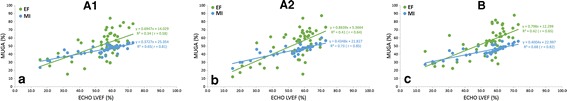
Linear least-squares fits of MUGA LVEF versus Echo LVEF and MUGA MI versus Echo LVEF by **a** A1, **b** A2, **c** B

**Fig. 3:**
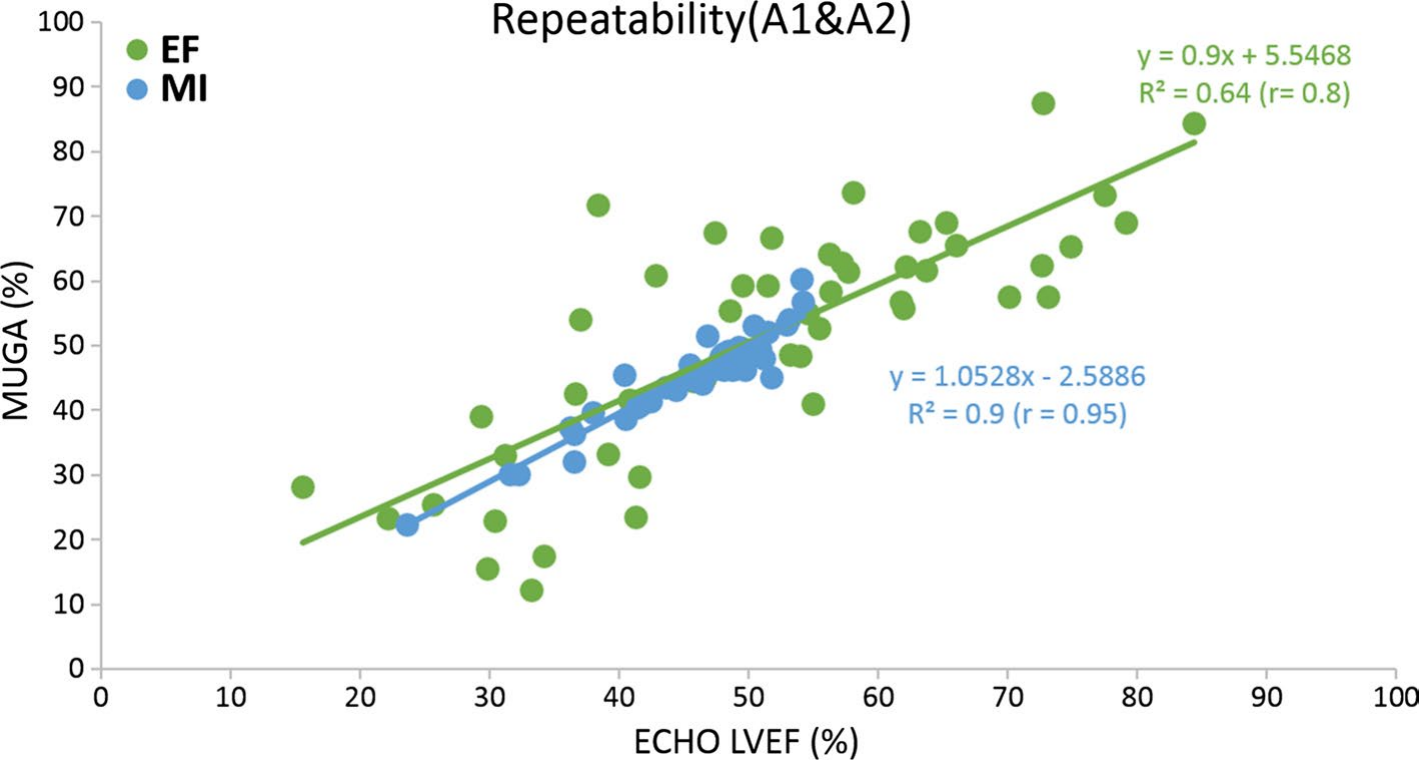
The linear least-squares fits of A1 versus A2 for MUGA LVEF and MUGA MI in terms of repeatability comparison

**Fig. 4:**
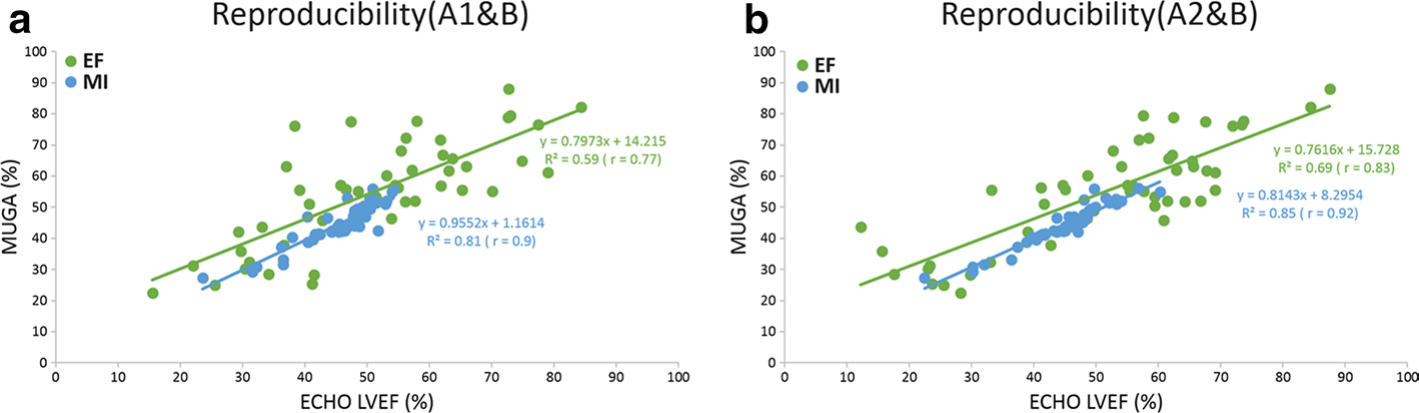
The linear least-squares fits of **a** A1 versus B, **b** A2 versus B for MUGA LVEF and MUGA MI showing correlation of LVEF assessments between operators. The reproducibility can be observed by compared of the r from MUGA LVEF and MUGA MI

**Fig. 5:**
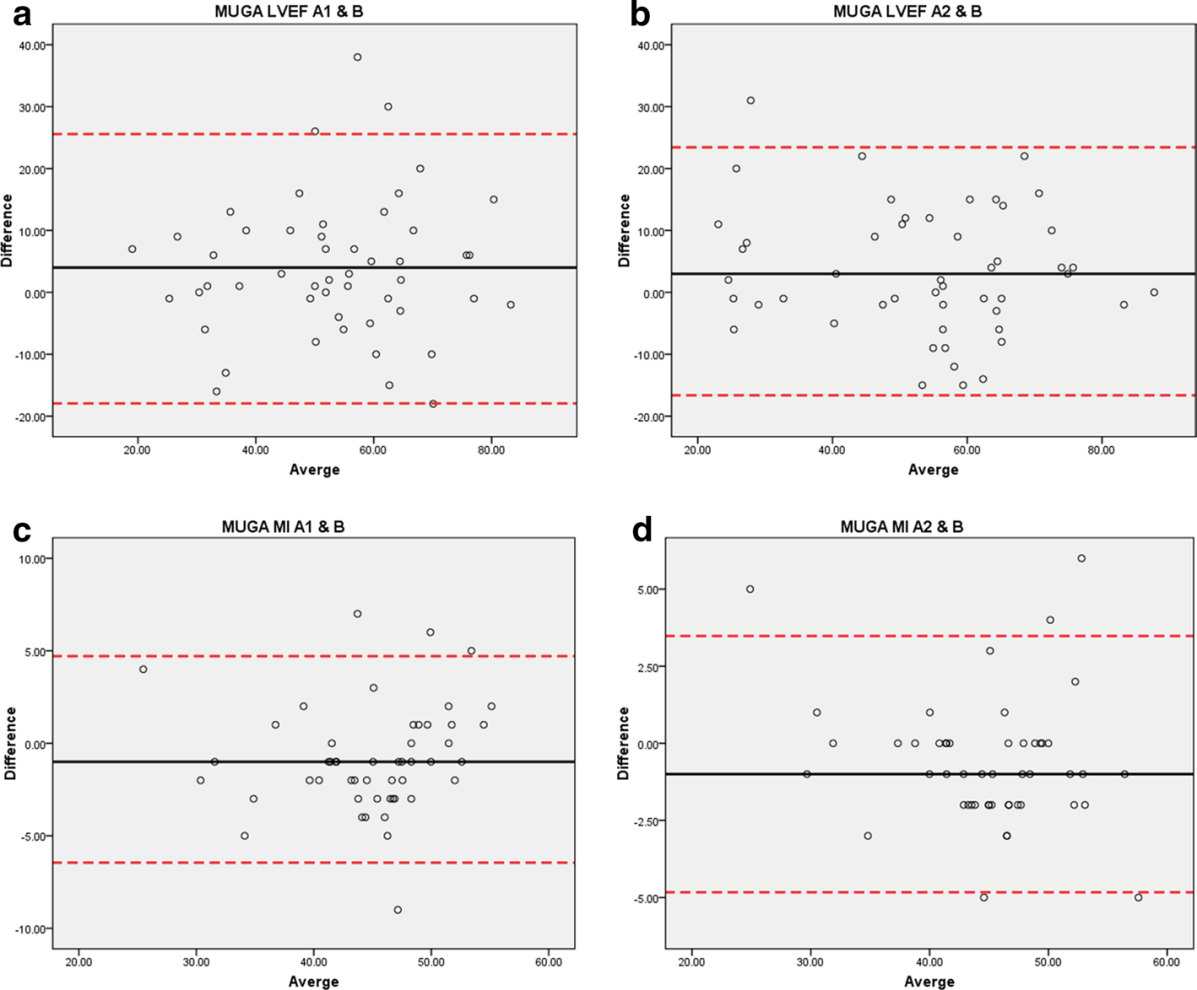
The Bland–Altman analysis of **a** A1 versus B, **b** A2 versus B for MUGA LVEF and **c** A1 versus B, **d** A2 versus B for MUGA MI showing consistency of LVEF assessments between operators

The mean LVEF was 53 ± 16 % by the MUGA LVEF method and 54 ± 13 % by the ECHO method respectively, while it was 45 ± 6 % by the MUGA MI method for all operations. MUGA MI estimation difference was statistically significant compared to the conventional MUGA LVEF and ECHO LVEF estimations (p < 0.005). ESV, EDV, and mean differences for the comparisons of MUGA MI and MUGA LVEF versus ECHO LVEF with the ROIs drawn by physician A (A1), the repeated drawing (A2) and physician B are listed in Table 1. Figure 2 shows linear least-squares fits of MUGA LVEF versus Echo LVEF by (a) A1 (b) A2 (c) B with correlation coefficient (r) 0.58, 0.64, 0.65 and MUGA MI versus Echo LVEF by (a) A1 (b) A2 (c) B with correlation coefficient (r) 0.81, 0.85, 0.82. The higher correlation coefficients (r) were obtained between proposed MUGA MI and ECHO LVEF compared to that between the conventional MUGA LVEF and ECHO LVEF. The linear least-squares fits of A1 versus A2 for MUGA LVEF and MUGA MI are plotted in Fig. 3. The higher repeatability for MUGA MI can be observed in the figure by the higher correlation coefficient for MUGA MI (r = 0.95) compared with that of MUGA LVEF (r = 0.80). Figure 4 shows (a) A1 versus B, (b) A2 versus B for MUGA LVEF and MUGA MI, respectively. Again, the reproducibility was better for MUGA MI (r = 0.90, 0.92 between A1 and B, A2 and B) than MUGA LVEF (r = 0.77, 0.83 between A1 and B A2 and B). The one way ANOVA was performed as well for repeatability and reproducibility. In MUGA MI estimation, there is no statistically significant difference between each dataset between A1 and A2 (p > 0.05), A1 and B (p > 0.05), which also represents the high repeatability and reproducibility. The Bland–Altman analysis and the intra-class correlation were presented in Fig. 5 and in Table 2, respectively. In Fig. 5, the solid (black) lines are the average difference between the involved two data sets; the dashed (red) lines represent the 95 % confidence regions. Figure 5a, b show the variation between different operators with the MUGA LVEF method while (c) and (d) show that with the MUGA MI method. One may notice that the average difference lines in (c) and (d) are close to 0 and the 95 % confidence regions are much smaller than that in (a) and (b), meaning the variation between different operators is much smaller for the MUGA MI method than the MUGA LVEF method. Both analyses show better conformity within MUGA MI than that within MUGA LVEF.

**Table 1:** ESV, EDV, mean difference in the comparisons of LVEF MI and MUGA LVEF versus ECHO LVEF with the ROIs drawn by the A1, A2 and B physicians

ROI	ESV^a^ (cm^2^)	EDV^a^ (cm^2^)	Mean difference in MUGA MI (%) vs ECHO LVEF (%)	Mean difference in MUGA LVEF (%) vs ECHO LVEF (%)
	Mean ± SD	Mean ± SD	Mean ± SD	Mean ± SD
A1	1.56 ± 0.61	2.66 ± 0.78	21.98 ± 12.50	23.85 ± 21.00
A2	1.63 ± 0.70	2.77 ± 0.78	21.42 ± 10.16	20.74 ± 18.56
B	1.66 ± 0.64	2.83 ± 0.73	23.34 ± 12.07	20.91 ± 30.59

^a^Left ventricle on planar views

**Table 2:** Mean values and intra-class correlations for LVEF MI and MUGA with the ROIs drawn by the A1, A2 and B physicians

	MUGA LVEF (Mean ± SD)	MUGA MI (Mean ± SD)
A1	51 ± 16	45 ± 6
A2	52 ± 18	45 ± 7
B	55 ± 17	44 ± 7
ICC (Inter-observer variability)	0.79	0.94
ICC (Intra-observer variability)	0.87	0.95

## Discussion

To the best of our knowledge, the presented MUGA MI method is the first non-linear approach for LVEF estimation using MUGA images. The major finding in this study was that LVEF MI value using MUGA image was correlated well with assessment of ECHO LVEF (r > 0.80) in comparison of low correlation from MUGA LVEF (r = 0.60). In previous studies, LVEF measurements by various techniques are not interchangeable [6, 11]. It is important to know whether the results of each technique are interchangeable, and thereby how the results of large studies in heart condition utilizing one technique can be applied using another. With the high correlation using MUGA MI to ECHO LVEFs, the MUGA MI becomes a potential bridge to transfer the LVEF from MUGA to ECHO. With complementary LVEF information from two different modalities, LVEF is more valuable in diagnosis, prognosis and therapeutic implications for patients suffering from left ventricular dysfunction. In clinical, if a patient with an ejection fraction close to the cutting edge or within the range between 40 and 60 % by MUGA LVEF, a second method could be used to provide more confidence in the estimation.

The inter- and intra-observer variations have been observed in previous studies, which is the clinical challenge when using the MUGA for LVEF measurement [6, 12, 13]. Bellenger et al. reported that the standard deviation was 58 % for MUGA vs ECHO for LVEF measurements as compared with each other [6]. The study of 20 patients with varying cardiac function who underwent repeat MUGA and echo imaging investigations was presented by Fletcher [12]. Hiscock et al. have investigated the LVEF measurement using MUGA image at 11 hospitals and the significant difference was found between the different processing workstations [13]. In the presented study, MUGA MI for LVEF estimation was calculated by the statistical dependence with non-linear approach based on histogram of two images. Therefore, the influences of LVEF measurement using MUGA such as low count density and incorrect background subtraction would be avoided in LVEF MI estimation in contrast to the linear LVEF calculation by the conventional MUGA approach. The non-linear analysis based on the histogram is not heavily dependent on the variation of the ROI delineation and noise background. However, very high noise in ROIs (left ventricle or heart background) can introduce variations on the sides of the histogram, which is derived from the ESV and EDV delineated by the operator, and consequently introduce some MI and thus LVEF errors. Our results demonstrate the consistent high correlation coefficients which were observed in repeated process (A1 and A2) and two independent operators (A, B) shown in Fig. 3. Using MUGA MI as estimation of LVEF in MUGA has the potential in variation reduction of inter-operator and intra-operator with high repeatability and reproducibility.

There are very wide variances ejection fraction between technologies, which are most marked in comparisons using MUGA and ECHO [6]. Our results agree with the previous study by looking the mean difference in MUGA LVEF versus ECHO LVEF as shown in Table 1. The standard deviation of mean difference was about 90 % variation to the mean difference between MUGA LVEF and ECHO LVEF. On the other hand, the standard deviation of mean difference was significant reduced in the comparison between MUGA MI and ECHO LVEF (Table 1). Although the standard deviation of mean difference between MUGA MI and ECHO LVEF was largely reduced, the high relative standard deviation to mean difference in the comparison between MUGA MI and ECHO LVEF (about 50 %) was still found in our result. Several factors caused the errors of LVEF measurements between ECHO and MUGA. First, MUGA image suffers from poor resolution, the need for background correction and errors from overlapping structures. In addition, ECHO LVEF extrapolates data from a limited sampling of the left ventricle, which makes the echo unreliable in the presence of regional asynergy, as it assumes that the area where the echo measurements are taken represents the entire left ventricle. Echo also suffers from errors introduced by gain-dependent edge identification and transducer position during imaging. These sources of error may contribute to the difference between Echo and MUGA. Also, the reproducibility and accuracy of conventional MUGA LVEF is dependent on the method used to identify and delineate end diastolic, end systolic and background regions. The variability in MUGA LVEF identified could be due, in part, to the processing method used in this study. Furthermore, the respiratory and cardiac motions were taken account into the LV volume delineation for both MUGA and ECHO imaging. The use of gated myocardial perfusion SPECT to assess left ventricular function and perfusion can be improved by using registration to remove left ventricular motion to allow perfusion image to be visualized in a static coordinate system [13]. An automatic alignment tool to improve repeatability of LV function in MUGA images was reported by Zhou et al. [14]. Slomka et al. applied thin plate splines to match all phases into the end-diastolic phase and to improve the effective resolution of the technique by removing motion-related blur [15]. One major concern in this study was that the motion remains a problem in MUGA imaging, due to motion correction was not performed in the present study. Thus, concatenating the registration into the LVEF MI estimation is being further investigated in our ongoing study.

A few limitations in this study are summarized here. Although the non-linear method introduced in this study can avoid dependence on the ROI delineation and noise background, very high noise in ROI can still add variations on both sides of the histogram, which may cause errors in LVEF estimation. Additionally, as gated MUGA images were acquired, if with image registration applied before data analysis, the motion blur problem due to respiration and heartbeat will improve. Finally, the major limitation in this study was the absence of comparison of MUGA MI with a ground truth. In this study, MUGA MI and MUGA LVEF were only compared with ECHO but the estimation of ECHO was highly operator dependent. Although our results show a closer correlation between ECHO and MUGA MI than ECHO and MUGA LVEF, it does not mean that MUGA MI is necessarily more accurate or reliable than MUGA LVEF. Therefore, an alternative method such as cardiac magnetic resonance imaging or Simpson method of ECHO, as the gold standard could be used to provide more confidence in the estimation. However, MUGA MI could be used to improve confidence when ECHO and MUGA are both performed for the LEVF estimation.

## Conclusions

In this study, our results demonstrated lower inter-observer variation and better repeatability by using MI for LVEF estimation on MUGA image compared to the conventional approaches on MUGA image. MUGA image with the aid of MI is promising to be more interchangeable LVEF to ECHO LVEF measurement.

## Abbreviations

LVEF: left ventricular ejection fraction
MUGA: multigated acquisition
ECHO: echocardiography
MI: mutual information
ECG: electrocardiographically
SPECT: single-photon emission tomography
LV: left ventricle
MRI: magnetic resonance imaging
EDV: end-diastolic volume
ESV: end-systolic volume
MUGA LVEF %: percentage of LVEF
RBCs: red blood cells
ROI: region of interest
